# Intra- and Intermolecular Hydrogen Bonding in Miscible Blends of CO_2_/Epoxy Cyclohexene Copolymer with Poly(Vinyl Phenol)

**DOI:** 10.3390/ijms23137018

**Published:** 2022-06-24

**Authors:** Wei-Ting Du, Yen-Ling Kuan, Shiao-Wei Kuo

**Affiliations:** 1 Department of Materials and Optoelectronic Science, Center for Functional Polymers and Supramolecular Materials, National Sun Yat-Sen University, Kaohsiung 80424, Taiwan; d093100002@student.nsysu.edu.tw (W.-T.D.); m103100012@nsysu.edu.tw (Y.-L.K.); 2 Department of Medicinal and Applied Chemistry, Kaohsiung Medical University, Kaohsiung 80708, Taiwan

**Keywords:** polycarbonate, CO_2_, alternating copolymers, H-bonding interactions, 2D-FTIR spectroscopy

## Abstract

In this study, we synthesized a poly(cyclohexene carbonate) (PCHC) through alternative ring-opening copolymerization of CO_2_ with cyclohexene oxide (CHO) mediated by a binary LZn_2_OAc_2_ catalyst at a mild temperature. A two-dimensional Fourier transform infrared (2D FTIR) spectroscopy indicated that strong intramolecular [C–H**···**O=C] hydrogen bonding (H-bonding) occurred in the PCHC copolymer, thereby weakening its intermolecular interactions and making it difficult to form miscible blends with other polymers. Nevertheless, blends of PCHC with poly(vinyl phenol) (PVPh), a strong hydrogen bond donor, were miscible because intermolecular H-bonding formed between the PCHC C=O units and the PVPh OH units, as evidenced through solid state NMR and one-dimensional and 2D FTIR spectroscopic analyses. Because the intermolecular H-bonding in the PCHC/PVPh binary blends were relatively weak, a negative deviation from linearity occurred in the glass transition temperatures (*T*_g_). We measured a single proton spin-lattice relaxation time from solid state NMR spectra recorded in the rotating frame [*T*_1ρ_(H)], indicating full miscibility on the order of 2–3 nm; nevertheless, the relaxation time exhibited a positive deviation from linearity, indicating that the hydrogen bonding interactions were weak, and that the flexibility of the main chain was possibly responsible for the negative deviation in the values of *T*_g_.

## 1. Introduction

Carbon dioxide (CO_2_) is a major contributor to the greenhouse effect that is inducing global warming. Possible ways to minimize the amount of CO_2_ in the atmosphere include a direct decrease in CO_2_ emissions, CO_2_ storage and capture, and chemical conversion of CO_2_. Especially, CO_2_ is the relatively nontoxic, naturally abundant, and inexpensive gas [1,2,3,4,5,6]. The use of CO_2_ as a feedstock could facilitate the large-scale syntheses of totally biodegradable copolymers, including polycarbonates and corresponding copolymers [7,8,9,10,11,12]. Inoue et al. were the first to report the green reactions of CO_2_ with epoxides [e.g., propylene oxide (PO), isobutylene oxide (IBO), and cyclohexene oxide (CHO)] to produce biodegradable polycarbonates [13,14]. For example, the alternating ring-opening copolymerization of CO_2_ with PO forms poly(propylene carbonate) (PPC), a major biodegradable polymer produced by the chemical industry with potential to mitigate the greenhouse effect [15,16].

Unfortunately, PPC is an amorphous material having a low glass transition temperature (*T*_g_) and poor mechanical and dimensional stability, displaying intrinsic brittleness. To improve its mechanical and thermal properties [*T*_g_ and thermal degradation temperature, (*T*_d_)], attempts have been made to increase its molecular weight through terpolymerization with other monomers (e.g., CHO), or through blending with other inexpensive polymers [e.g., ethylene–*co*–vinyl alcohol (EVOH), poly(methyl methacrylate) (PMMA), poly(lactic acid) (PLA), starch, and cellulose] [17,18,19,20,21,22]. Nevertheless, because of the low miscibility of PPC with these polymers, improvements in the values of *T*_g_ and mechanical properties have been unsatisfactory in most cases.

The chemical structure of PPC features two electron-rich moieties: C–O–C and C=O units; thus, secondary intermolecular forces (e.g., H-bonding interactions) can enhance its thermal and mechanical properties upon blending with H-bond-donating polymers [e.g., poly(vinyl phenol) (PVPh), poly(vinyl alcohol), and bisphenol A] [23,24,25,26]. The H-bonding interaction in PPC/PVPh blends have been characterized using FTIR, X-ray photoelectron, electron spin resonance, and Raman spectroscopy [25,26], confirming that H-bonding between the PPC C=O units and PVPh OH units enhances the miscibility of this binary blend. Nevertheless, compared to the H-bonding in binary blends of PVPh with other carbonyl-functionalized polymers (e.g., polyvinylpyrrolidone, polycaprolactone, and even PMMA) [27,28,29,30,31,32,33], a relatively lower fraction of H-bonded C=O units appear in the PPC/PVPh binary blends. This phenomenon is similar to that in PLA/PVPh binary blends, where strong intramolecular [C–H···O=C] H-bonding in PLA homopolymers inhibits the intermolecular H-bonding of the OH units of PVPh with the C=O units of PLA, where the interassociation equilibrium constant is quite low (*K*_A_ < 10), indicative of a low ability to accept H-bonding [33,34,35]. Detailed investigations of H-bonding between CO_2_-based polycarbonates and other polymeric materials remains a challenge, but such studies would likely improve the miscibility and thermal and mechanical properties of CO_2_-based polycarbonates.

In this study, we chose poly(cyclohexene carbonate) (PCHC) as a model polycarbonate for blending with homopolymeric PVPh, because the value of *T*_g_ of PCHC (ca. 108 °C) is close to that of PVPh (*T*_g_ = 168 °C in this study). Such a small difference in the values of *T*_g_ in a weakly hydrogen bonded binary blend can avoid the occurrence of dynamically heterogeneous properties that can arise when two values of *T*_g_ appear during dynamic scanning calorimetry (DSC) [36,37,38,39]. We synthesized PCHC through alternative copolymerization of CO_2_ with CHO, using LZn_2_OAc_2_ as a binary catalyst, at a mild temperature. We then formed binary blends of PCHC and PVPh through solvent casting in tetrahydrofuran (THF) solutions. Finally, we used DSC, one-dimensional (1D) and two-dimensional (2D) FTIR spectroscopy, and solid-state NMR spectroscopy to characterize the miscibility, H-bonding interaction, and domain size of PVPh/PCHC binary blends.

## 2. Results and Discussion

### 2.1. Synthesis of LH and LZn_2_OAc_2_

LH, synthesized by 2,2-dimethyl-1,3-propanediamine and 2 equivalents of *o*-vanillin, was characterized by FTIR and NMR spectroscopy shown on Figure S2. The signal of the hydroxyl group was at 3450 cm^−1^ and the signal of the imine group was at 1632 cm^−1^, overlapped with the C–H stretching of an aromatic ring. Figure S2c displays the integral ratio of methoxy group and methyl group as 1:1, which means we obtained LH successfully.Afterward, we synthesized LZn_2_OAc_2_ by LH and Zn(OAc)_2_·2(H_2_O) with two equivalents. In Figure S2b, the signal of imine group shifted to 1624 cm^−1^ and C=O of carboxyl shifted to 1637 cm^−1^, relatively, due to the coordination with zinc. The broad signal at 3200 cm^−1^ was from moisture. In addition, there was further evidence for the coordination with zinc. The signal of hydroxy at 14.15 ppm disappeared in ^1^H NMR of LZn_2_OAc_2_. Furthermore, the integral ratio of acetate and methoxy was 1:1, which means the catalyst was dinuclear. This synthesis was performed based on the report from the Williams group [40].

### 2.2. Synthesis of PCHC

**Scheme 1:**
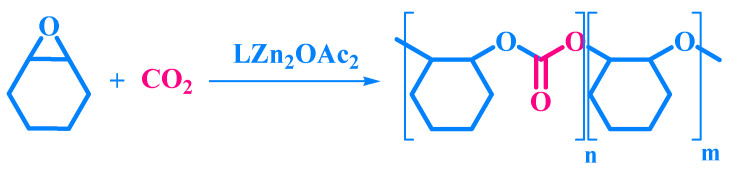
Synthesis of the PCHC copolymer.

We synthesized PCHC through the copolymerization of CO_2_ with CHO, mediated by the catalyst LZn_2_(OAc)_2_ as shown in Scheme 1 and characterized its chemical structure and composition using FTIR, MALDI-TOF mass, and NMR spectroscopy. 

**Figure 1:**
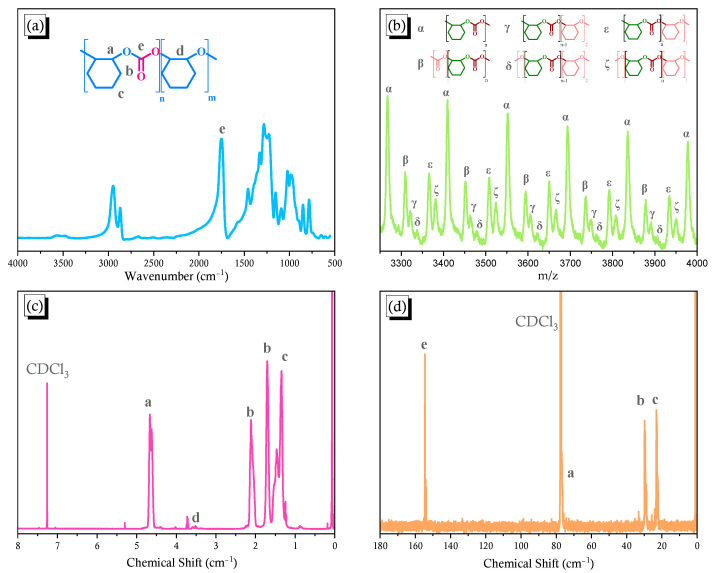
(**a**) Synthesis of the PCHC copolymer and its FTIR, (**b**) MALDI-TOF mass, (**c**) ^1^H NMR, and (**d**) ^13^C NMR spectra.

Figure 1a presents the FTIR spectrum of the pure PCHC, revealing a strong C=O absorption at 1751 cm^–1^, consistent with a linear carbonate copolymer, rather than a cyclic carbonate monomer [40,41]. To investigate the copolymer compositions in the pure PCHC, we recorded MALDI-TOF mass spectra (Figure 1b) [42,43,44]. The difference between two signals at *m*/*z* 3266.98 and 3409.57 was ca. 142 g mol^–1^, which is equal to the summed molecular weights (*M*_w_) of one CHO and one CO_2_ unit; the signal at *m*/*z* 3266.98 represented 23 cyclohexene carbonate units. Similarly, the difference at *m*/*z* 3266.98 (α) and 3310.62 (β) was ca. 44 g mol^–1^, equal to the value of *M*_w_ of a CO_2_ unit. Furthermore, a few polyether units were present in the copolymer, as evidenced by the difference at *m*/*z* 3266.98 (α) and 3365.56 (ε) being approximately 98 g mol^–1^, which is equal to the value of *M*_w_ of one CHO unit, and the signal at *m*/*z* 3382.06 (ξ) representing the copolymer having one more terminal oxygen atom than that giving the signal at *m*/*z* 3365.56 (ε). In addition, the signal at *m*/*z* 3320.9 (γ) represents a copolymer having two more CHO units and one less cyclohexene carbonate unit than that of the copolymer providing the signal at *m*/*z* 3266.98 (α); the low intensity of these signals suggested a low polyether content in the copolymer. In addition, the signal at *m*/*z* 3337.28 (δ) suggested one more oxygen atom at the end of the copolymer than that in the copolymer giving the signal at *m*/*z* 3320.9 (γ). ^1^H and ^13^C NMR spectra of the PCHC copolymer were as displayed in Figure 1c,d. Signals appeared in ^1^H NMR spectrum at 1.34, 1.70, and 2.11 ppm for the cyclohexyl CH_2_ units on the side chains, and at 4.66 ppm for the cyclohexyl CH units on the main chain [45]. A signal for polyether forms of the cyclohexyl CH groups on the main chain appeared at 3.58 ppm. The calculated content of poly(cyclohexene oxide) was only approximately 2.2% in the copolymer, meaning that its content was negligible. Signals appeared in the ^13^C NMR spectra at 23.18 and 29.84 ppm for the cyclohexyl **C**H_2_ units on the side chains, at 76.86 ppm for the cyclohexyl **C**H units on the main chain, and at 154.59 ppm for the C=O carbon nuclei, providing direct evidence for the PCHC copolymer containing CO_2_ units. Gel permeation chromatography [GPC, Figure S1a] revealed that the molecular weight of this PCHC copolymer (*M*_n_) was 21,900 g mol^–1^ (PDI = 1.72).

**Figure 2:**
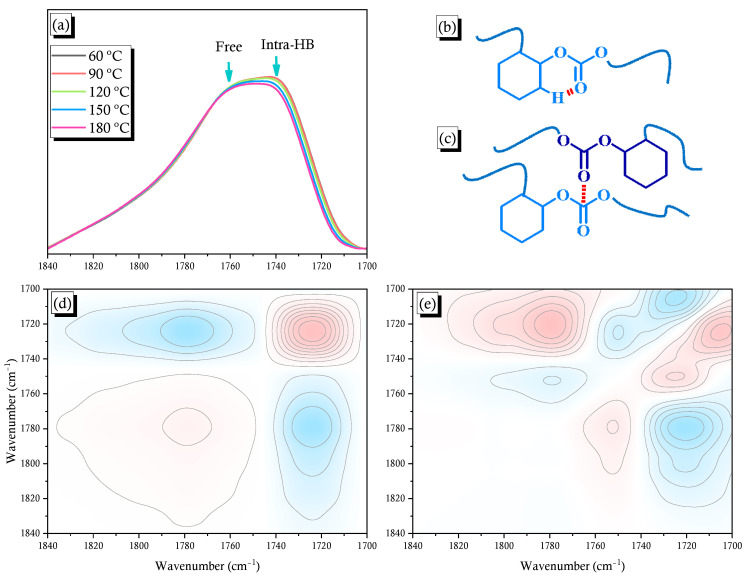
(**a**) FTIR spectra of the pure PCHC copolymer, recorded at various temperatures; (**b**) Schematic representation of the intramolecular [C–H**···**O=C] H-bonding; (**c**) Schematic representation of the free C=O units undergoing dipole–dipole interactions; (**d**,**e**) 2D FTIR spectral (**d**) synchronous and (**e**) asynchronous correlation maps.

A unique C=O peak appeared in the FTIR spectrum of the pure PCHC in Figure 1a. The full widths at half maximum (FWHM) of this signal in the spectrum of PCHC (ca. 63 cm^–1^) was much broader than those for pure PMMA (ca. 38 cm^–1^) and poly(vinyl pyrrolidone) (PVP; ca. 46 cm^–1^) [27,43,44]. Previous studies have revealed that the C=O bands of pure PMMA and pure PVP homopolymers become broader as a result of dipole–dipole interaction of their C=O groups. To confirm whether any specific interactions exist for the pure PCHC, we recorded its temperature-dependent FTIR spectra (Figure 2a). Two main minima peaks appeared at 1763 and 1735 cm^–1^ (Figure S3), based on the second-derivative spectra from Figure 2a, corresponding to C=O groups free from dipole–dipole interactions (Figure 2c) and intramolecularly H-bonded [C–H**···**O=C] units in six-membered rings (Figure 2b), respectively. Furthermore, the fraction of intramolecular H-bonded [C–H**···**O=C] units decreased upon increasing the temperature (Figure 2a), as expected. Ozaki et al. reported that the C=O units in the PLA main chain do not experience strong H-bonding, but rather dipole–dipole interactions and weak [C–H**···**O=C] H-bonding [46]. Nevertheless, experimental data for this kind of weak [C–H**···**O=C] H-bonding is difficult to measure because such interactions generally coexist with other stronger types of H-bonding.

2D IR correlation spectroscopy can be used to investigate the strengths of various intra- and intermolecular interactions based on 1D IR spectra through analyses of selected absorption bands. This novel approach has been used extensively in polymer science to monitor specific interactions in response to spectral perturbations resulting from changes in temperature, time, composition, and pressure [47,48,49,50]. In this study, positive and negative cross-peaks are presented in red and blue areas, respectively, of the 2D IR correlation contour map. We recorded both synchronous and asynchronous correlational maps of the 2D FTIR spectra. The 2D synchronous spectrum was symmetrical on the correlation map with respect to the diagonal line. This auto peak represented the degree of autocorrelation arising from the perturbations induced by molecular vibration; it was located at the diagonal position in the synchronous 2D spectra. If any auto peaks were present, signals whose values were always positive at the corresponding wavenumbers would vary under environmental perturbation greatly. The cross-peak was located at the off-diagonal position of the synchronous 2D spectra; the signal could be positive or negative, corresponding to the concurrent or converse change of the spectral intensity variation located at ν_1_ or ν_2_. A positive cross-peak would imply that the intensity fluctuations of the two peaks at ν_1_ and ν_2_ under perturbation had occurred at the same direction (such as both decreasing or both increasing). A negative cross-peak would indicate that the intensity of the two peaks at ν_1_ and ν_2_ under environmental perturbation would vary in opposite directions (such as one would increase and the other would decrease) [51]. As in most synchronous spectra, any asynchronous cross-peak signal could also be either positive or negative. Such signals would offer useful spectroscopic information regarding the sequential order in response to the external stimulus. The 2D asynchronous spectra were asymmetric in the correlation map with respect to the diagonal lines. Following Noda’s rule [51], if the signal of the cross-peaks in the synchronous or asynchronous correlation maps was the same (both red or blue), then the ν_1_ band will vary prior to the ν_2_ band. In contrast, if the signals of the cross-peaks are different in the synchronous or asynchronous spectra (one red and the other blue) under perturbation, the ν_1_ band will vary after the ν_2_ band.

Figure 2d presents the synchronous 2D IR correlation map for the C=O groups at 1700–1840 cm^–1^, based on Figure 2a. The signal for the free C=O unit in PCHC located at 1763 cm^–1^ and that for intramolecularly H-bonded C=O unit located at 1735 cm^–1^. Negative cross-peaks appeared for the signals at 1735 and 1763 cm^–1^, suggesting that these two absorption bands varied in opposite directions: one is an H-bonded functional group and the other is a free C=O group, as expected. The FTIR spectral signal of a free C=O group often appears at a wavenumber higher than that of an H-bonded C=O group, because the more obvious charge distribution can lead to a stronger IR vibration. The asynchronous 2D correlation map in Figure 2e reveals negative cross-peaks in the lower right (1763 vs. 1735 cm^–1^) exhibiting the same behavior as the synchronous map; thus, the peak at 1735 cm^–1^ was altered prior to the peak at 1763 cm^–1^. This phenomenon is reasonable because an H-bonding interaction is more sensitive to an increase in temperature than is a free C=O group, thereby resulting in a later response for a free C=O group. To the best of our knowledge, this is the first study to use a combination of 1D and 2D FTIR spectroscopic analyses to investigate these types of specific interactions in CO_2_-based copolymers. We conclude that strong intramolecular [C–H**···**O=C] interactions existed in the PCHC copolymer, thereby inhibiting this copolymer’s ability to interact intermolecularly with other polymers. Therefore, it would be necessary to blend PCHC with a strongly hydrogen-bond-donating polymer (e.g., PVPh) to obtain miscibility through intermolecular H-bonding (e.g., with the OH groups of PVPh, as in this study).

### 2.3. PCHC/PVPh Binary Blends

**Figure 3:**
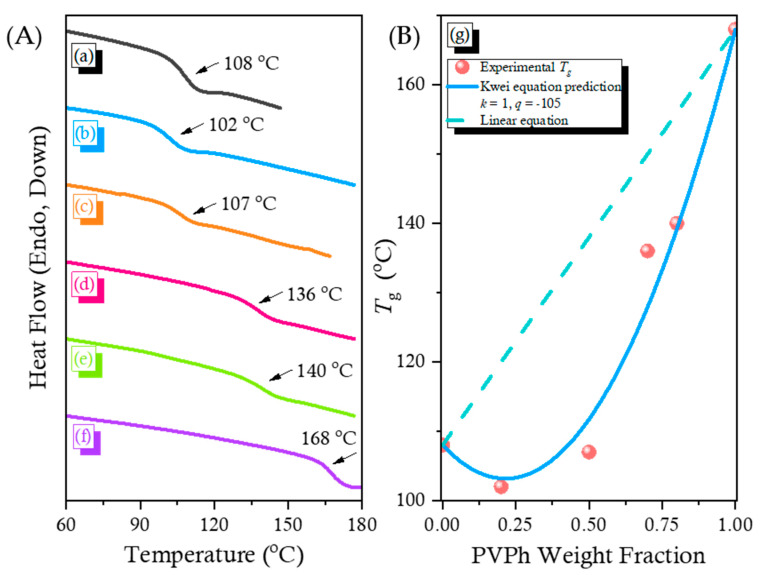
(**A**) DSC thermal analyses of PVPh/PCHC binary blends: (a) pure PCHC, (b) PCHC-80, (c) PCHC-50, (d) PCHC-30, (e) PCHC-20, and (f) pure PVPh; (**B**): (g) Corresponding values of *T*_g_ predicted by the linear rule and the Kwei equation.

DSC thermal analysis is a general characterization for measuring the miscibility of binary polymer blends. Figure 3A, a–f present the DSC traces of the pure PCHC, the pure PVPh, and their corresponding binary blends. The single values of *T*_g_ indicate that no macro-phase separation was occurring and imply that the blends exhibited miscible behavior. Furthermore, upon increasing the concentration of PVPh, the value of *T*_g_ decreased slightly initially, but then increased dramatically. Among the many equations proposed for modifying the Fox equation to describe the composition-dependence of values of *T*_g_, the Kwei equation is usually used for miscible polymer blend systems that feature specific interactions [52]:

(1) $$T_{g}=\frac{W_{1}T_{g1}+kW_{2}T_{g2}}{W_{1}+kW_{2}}+qW_{1}W_{2}$$

where *W*_1_ or *W*_2_ represent weight fraction of each polymer; *T*_g1_ or *T*_g2_ is the glass transition temperature of PCHC and PVPh, respectively; *k* and *q* are the fitting constant. The value of *q* is generally corresponding to the H-bonding strength of the miscible polymer blend. The values of *k* and *q* of 1 and −105 are obtained, respectively, from the Kwei equation (Figure 3B, g). The linear rule could not fit the binary PVPh/PCHC blend system well. As a result, we obtained a negative value of *q*, suggesting that intermolecular H-bonding in the PVPh/PCHC blend was weaker than the self-association H-bonding of pure PVPh [33].

**Figure 4:**
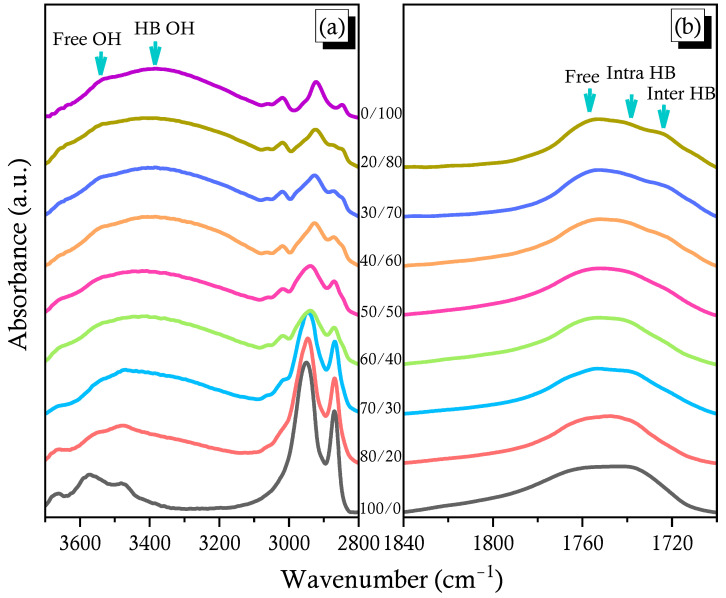
FTIR spectra of the various PVPh/PCHC binary blends, recorded at 120 °C: (**a**) OH and (**b**) C=O stretching regions.

IR spectroscopy is a general method for investigating polymeric interactions occurring in the solid state, both qualitatively and quantitatively [33]. Figure 4a shows the FTIR spectral regions representing the OH stretching absorptions of the pure PCHC, the pure PVPh, and various PVPh/PCHC blends, measured at 120 °C to avoid the effects of absorbed moisture. The signal for end-OH stretching of PCHC appeared near 3670 cm^–1^; the signals at 3575 and 3470 cm^–1^ are overtones of the signals for free and intramolecularly H-bonded C=O groups, respectively. The OH stretching band of PVPh extended from 3100 to 3600 cm^–1^, corresponding to the signals of the free OH units at 3540 cm^–1^ and of the self-associated OH**···**OH units at 3385 cm^–1^. The intensities of the signals for both free and self-associated OH units of PVPh both decreased upon increasing the concentration of PCHC; furthermore, the signal for the self-associated OH units was shifted to a higher wavenumber (ca. 3446 cm^–1^) when compared with that of the pure PVPh, representing a transformation to relatively weak intermolecular OH**···**O=C H-bonds in the PVPh/PCHC binary blends [33].

**Figure 5:**
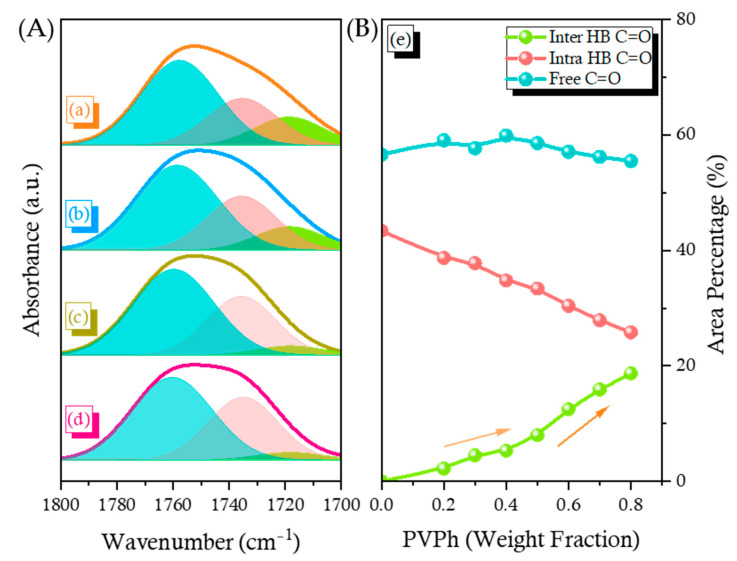
(**A**): (a–d) Curve fitting of the C=O absorptions of selected PVPh/PCHC binary blends, 30/70, 40/60, 60/40, 70/30, relatively; (**B**): (e) Area fractions of the free C=O, intramolecularly H-bonded C=O, and intermolecularly H-bonded C=O groups in PVPh/PCHC binary blends.

Figure 4b presents the corresponding FTIR spectra of the C=O regions, also measured at 120 °C. Three main minima peaks appeared at 1763, 1735, and 1719 cm^–1^ in Figure S4, based on the second-derivative spectra in Figure 4b. Because the signals for PCHC at 1763 and 1735 cm^–1^ represented free and intramolecularly H-bonded C=O units, respectively, we assigned the signal at 1719 cm^–1^ to the C=O units of PCHC interacting with the PVPh OH units through intermolecular H-bonding. The intensity of the absorption at 1719 cm^–1^ increased upon increasing the PVPh content in this binary blend; Figure 5A, a displays the selected curve fitting results for these three peaks for blends of PCHC-30, PCHC-40, PCHC-60, and PCHC-70. Figure 5A, b reveals that the area fraction of the intermolecularly H-bonded OH**···**O=C units of PCHC increased, while that of the intramolecularly H-bonded C–H···O=C units of PCHC decreased, upon increasing the concentration of PVPh, as expected. We calculated the *K*_A_ value (*K*_A_ = 5) for the PVPh/PCHC binary blend, as displayed in Figure S5, based on the Painter–Coleman association model; Table 1 summarizes the corresponding thermodynamic parameters [53]. This value of *K*_A_ is much smaller than the value of *K*_B_, indicating that the intermolecular H-bonding was weaker than that of the pure PVPh, consistent with the negative value of *q* we obtained using the Kwei equation [33]. In addition, the fraction of intermolecularly H-bonded C=O groups was higher than that predicted when the content of PVPh was greater than 50 wt%, consistent with the DSC data, where the value of *T*_g_ increased significantly as this composition (Figure 3B, g). We conclude that, when blending with the strong hydrogen bond donor PVPh, the blend composition should be greater than 50 wt% to enhance the fraction of intermolecular H-bonding, thereby obtaining a higher value of *T*_g_ for the PVPh/PCHC binary blends.

**Figure 6:**
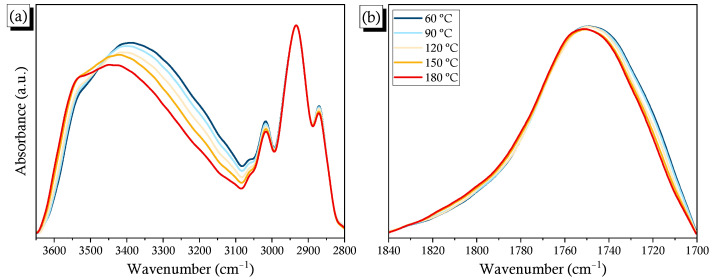

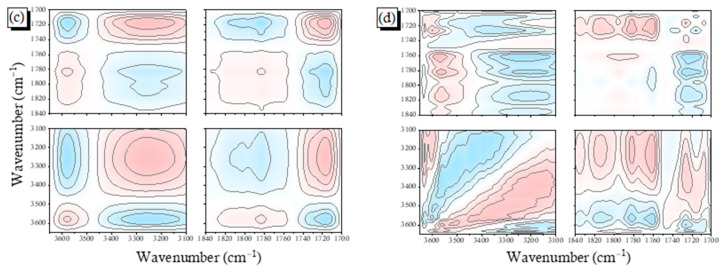
(**a**,**b**) FTIR spectra of the PCHC-50 binary blend, recorded at various temperatures: (**a**) OH and (**b**) C=O regions; (**c**,**d**) Corresponding 2D FTIR spectral; (**c**) synchronous; and (**d**) asynchronous correlation maps.

To confirm that H-bonding interactions were occurring in this binary blend, we examined whether the temperature influenced the intermolecular interactions between PCHC and PVPh. Figure 6a reveals that the intensity of free OH units increased, but that for the intermolecular H-bonded units decreased and shifted to higher wavenumber, upon increasing the temperature of the PCHC-50 blend. On the other hand, the signal for the C=O groups also shifted to higher wavenumber upon increasing the temperature, indicating that the strength of its intermolecular H-bonding also decreased (Figure 6b). 2D FTIR spectral analyses provided more effective information about the H-bonding interactions in this binary blend. In the synchronous 2D spectra (Figure 6c), the band of the free OH units appeared at 3670 cm^–1^ and that of the H-bonded OH units in the range 3100–3600 cm^–1^; these two bands displayed a negative correlation. Similar to the results in Figure S4, three bands appeared for C=O units in the 1D FTIR spectrum; nevertheless, the band for the free C=O units was negatively correlated to the bands for the intra- and intermolecularly H-bonded C=O groups, as expected. Furthermore, we also investigated the correlation between the OH and C=O groups. The band for the free OH groups exhibited a positive correlation with the band for the free C=O units; the band for the H-bonded OH units also displayed a positive correlation with the band for the H-bonded C=O units. In contrast, the band for the H-bonded OH units displayed a negative correlation with the band of the free C=O units, and the band for the free OH groups exhibited a negative correlation with the band for the H-bonded C=O units, as expected because of the opposite directions in which each band moved. Figure 6d displays the sequence of the variations in the H-bonded and free groups; the cross-peak between the signals at 3670 and 3250 cm^–1^ was negatively correlated, the same as the behavior in the synchronous spectrum, indicating that the perturbance affected the H-bonded OH groups prior to the free OH groups. Similarly, the H-bonded C=O groups were altered before the free C=O groups, while the intermolecularly H-bonded C=O groups were altered after the intramolecularly H-bonded C=O groups, due to the correlations being opposite to those in the synchronous 2D spectrum. In addition, the signal (3670 vs. 1763) was negative, whereas the signal (3250 vs. 1763) was positive, opposite to the behavior in the synchronous spectrum, suggesting that the free C=O groups were altered after the free and H-bonded OH groups. Furthermore, the signal for (3670 vs. 1719) was negative while the signal for (3250 vs. 1719) was positive, the same as the behavior in the synchronous spectrum, indicating that the perturbance affected the H-bonded C=O groups prior to the H-bonded and free OH groups. Taken together, the 2D maps revealed that the sequence for changes in intensity upon increasing the temperature followed the order intramolecularly H-bonded C=O (1735 cm^–1^) > intermolecularly H-bonded C=O (1719 cm^–1^) > H-bonded OH (3250 cm^–1^) > free OH (3670 cm^–1^) > free C=O (1763 cm^–1^). Thus, the intramolecularly H-bonded C=O groups were the first to undergo de-association; they then formed intermolecularly H-bonded C=O groups with the OH groups of PVPh. The free OH and C=O groups in the PVPh/PCHC binary blends were the last to be influenced upon increasing the temperature.

### 2.4. Solid State NMR Spectra

**Figure 7:**
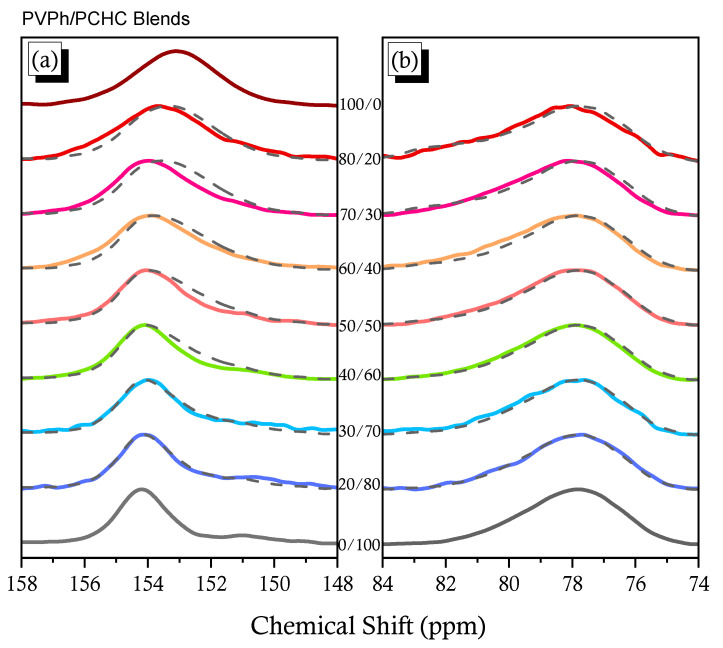
Selected carbon nuclei in solid state ^13^C NMR spectra: (**a**) C–OH and C=O carbon nuclei of PVPh and PCHC and (**b**) C–O carbon nuclei of PCHC.

Solid state NMR spectroscopy could provide insight into the H-bonding interaction, the domain size, and the mobility in this PVPh/PCHC binary blend (Figure S6). Figure 7 displays the signals of selected carbon nuclei of the pure PCHC, the pure PVPh, and their corresponding binary blends. The signal for the C=O carbon of the pure PCHC was located at δ = 154.2 ppm, the signal for the phenolic C–OH carbon of the pure PVPh was located at δ = 153.1 ppm, and the signal for the C–O carbon of the pure PCHC was located at δ = 77.8 ppm. Combining experimental data with simulated data can confirm the possible H-bonding interactions in PCHC/PVPh blend systems [54].

The simulated spectra of the blend systems (dashed lines), determined simply by summing the experimental data for the pure PCHC and pure PVPh at the relevant blend compositions, were very different from the experimental spectra of the PCHC blend systems. In Figure 7a, the experimental spectra featured complicated and broad signals, consistent with H-bonding between the phenolic OH units of PVPh and the C=O units of PCHC. Furthermore, Figure 7b reveals that the signal for the C–O carbon nuclei of PCHC shifted downfield slightly upon increasing the concentration of PVPh, the result of intermolecular H-bonding of the C=O groups.

**Figure 8:**
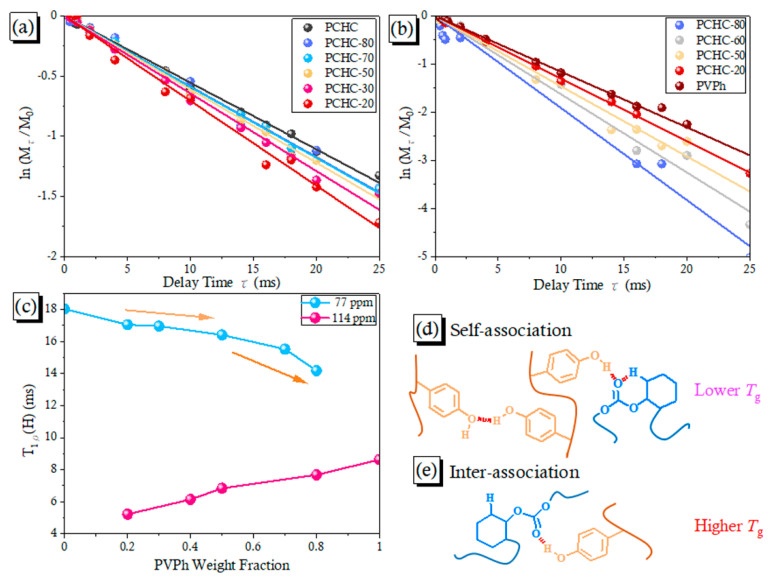
(**a**,**b**) Semi-logarithmic plots of the magnetization intensities of the signals at (**a**) 114 and (**b**) 77 ppm, plotted with respect to the delay time of various PVPh/PCHC binary blends; (**c**) Values of *T*_1ρ_(H) of various PVPh/PCHC binary blends; (**d**,**e**) Possible H-bonding through (**d**) self-association H-bonding of PVPh at lower concentrations of PVPh; and (**e**) intermolecular H-bonding of PVPh/PCHC binary blends at higher concentrations of PVPh.

The domain sizes and molecular mobility of H-bonded blends can be determined through measurements of the value of T_1__ρ_(H) in the rotating frame [55,56]; the value of *T*_1__ρ_(H) can be determined from *M_τ_* = *M_0_* exp(−τ ⁄ *T*_1__ρ_ (*H*)), where *τ* represents the spin-lock time and *M_τ_* and *M*_0_ are the experimental intensity measured after *τ* seconds and the intensity of peak at *t* = 0, respectively. Figure 8a,b display the plots of ln(*M_τ_*/*M*_0_) with respect to *τ* for the **C**–O units of PCHC at δ = 77 ppm and for the **C**–C–OH units of PVPh at δ = 114 ppm. The experimental results all displayed single-exponential-decay functions. A single value of T_1__ρ_(H) was calculated for the blend system, based on the slope of the plot, implying that the miscibility dimension of the blend system was short, based on the 1D diffusion equation, with an average diffusive path length of approximately 2–3 nm, depending on the average distance between two protons as well as their dipolar interactions. This PVPh/PCHC binary blend system could be taking into account for having one single component, and could be considered miscible from a thermodynamic viewpoint of such an H-bonding system. Figure 8c presents the values of T_1__ρ_(H) measured at 77 and 114 ppm. These values of T_1__ρ_(H) decreased upon increasing the other content, implying that the domain size decreased as a result of intermolecular H-bonding, with the most dramatic descent at 77 ppm occurring after 50 wt% of PVPh had been added, consistent with the sudden ascent in the value of *T*_g_ in Figure 3B, g. Thus, prior to the addition of 50 wt% of PVPh, most of the units in PCHC were involved in intramolecular H-bonding with free C=O groups present, as in Figure 2b, and most of the units in PVPh existed as self-association H-bonded OH groups, as in Figure 8d; upon increasing the PVPh concentration thereafter, an obvious increase in intermolecular H-bonding occurred, as in Figure 8e. Overall, the relaxation times displayed a positive deviation from linearity, indicating that the H-bonding interactions were weak, with possible flexibility of the main chain, resulting in negative deviations in the values of *T*_g_ when compared with the linear rule.

## 3. Materials and Methods

### 3.1. Materials

CHO (Acros Organic, Geel, Belgium) was heated under reflux over CaH_2_ for 1 day and vacuum-distilled prior to use. Zinc acetate dihydrate (98%) was purchased from SHOWA (Tokyo, Japan). PVPh [*M*_n_ = 24,200 g mol^–1^; PDI = 1.66; Figure S1b] was obtained from Sigma–Aldrich (St. Louis, MO, USA). High-purity CO_2_ (>99.999%) was purchased from Hsin E Li Gas Industrial (Kaohsiung, Taiwan). The synthesis of the LZn_2_(OAc)_2_ catalyst [40] is discussed in the Supporting Information (Figure S2).

### 3.2. Copolymerization of CO_2_ and CHO

LZn_2_(OAc)_2_ (1.1018 g) was introduced into an autoclave equipped with a stirring bar and a vacuum line was connected to the reaction system. The catalyst was dried under the vacuum at 100 °C for 8 h. After cooling, the autoclave was purged with CO_2_ and CHO (16 mL) was added. The copolymerization was performed in an oil bath at 80 °C for 20 h at a constant CO_2_ pressure of 435 psi. The reactor was cooled and the unreacted CO_2_ was released carefully. The mixture was dissolved in CH_2_Cl_2_ and extracted with 5% dilute hydrochloric acid. The solution was precipitated several times in MeOH and then the PCHC was dried in a vacuum oven at 60 °C. ^1^H NMR (500 MHz, CDCl_3_, δ, ppm): 4.66 (CyC**H**OCO_2_), 3.5 (CyC**H**OC), 1.26–2.26 (CyC**H**_2_). ^13^C NMR (125 MHz, CDCl_3_, δ, ppm): 154.5 (C=O), 76.8 (Cy**C**HOCO_2_), 21.9–30.7 (Cy**C**H_2_). FTIR (KBr, cm^–1^): 1751 (C=O). GPC [Figure S1a]: *M*_n_ = 21,900 g mol^–1^; PDI = 1.72.

### 3.3. PVPh/PCHC Binary Blends

Various binary PVPh/PCHC blends were prepared by using solution blending. Mixtures (5 wt%) in THF were stirred for 1 day. The solvent was evaporated slowly over 3 days at 40 °C and the residual solvent was gradually removed under high vacuum at 60 °C for 2 days.

## 4. Conclusions

We synthesized a PCHC alternating copolymer through ring-opening polymerization of CO_2_ and CHO monomers. We used FTIR spectroscopy, MALDI-TOF mass spectrometry, and NMR spectroscopy to characterize the chemical structure of this PCHC alternative copolymer. 2D FTIR spectral analyses revealed strong intramolecular [C–H···O=C] H-bonding of the PCHC copolymer. DSC thermal analyses indicated that the PVPh/PCHC binary blends featured a single value of *T*_g_ at each blend composition, suggesting fully miscible behavior, with solid state FTIR and NMR spectral analyses suggesting weak intermolecular H-bonding between the OH groups of PVPh and the C=O groups of PCHC. In addition, DSC analyses revealed a negative deviation in the values of *T*_g_, arising because of a lower *K*_A_/*K*_B_ ratio for this PVPh/PCHC binary blend; the relaxation times displayed a positive deviation from linearity, indicating that the H-bonding interactions were weak and possibly that the main chains were flexible.

## Figures and Tables

**Table 1:** The thermodynamic parameters of PVPh/PCHC binary blends at room temperature.

Polymer	Molar Volume (mL mol^–1^)	Molecular Weight (g mol^–1^)	Solubility Parameter (cal mL^–1^)	DP	Equilibrium Constants		
					*K*_2_	*K*_B_	*K*_A_
PVPh	100.0	120.0	10.6	201	21.0	66.8	–
PCHC	94.5	142.0	10.2	154	–	–	5

*K*_2_ and *K*_B_: self-association equilibrium constants; *K*_A_: interassociation equilibrium constant.

## Data Availability

Not applicable.

## Supplementary Materials

The following are available online at https://www.mdpi.com/article/10.3390/ijms23137018/s1.
