# Supplementary material for: Intra- and Intermolecular Hydrogen Bonding in Miscible Blends of CO2/Epoxy Cyclohexene Copolymer with Poly(Vinyl Phenol)
Source: Int J Mol Sci. 2022 Jun 24;23(13):7018. doi: 10.3390/ijms23137018 (PMC9266814; doi:10.3390/ijms23137018)
Supplement: Supplementary file 1 [file ijms-23-07018-s001.zip › ijms-1776837-supplementary.pdf]

**Supporting Information for**

# Intra- and Intermolecular Hydrogen Bonding in Miscible Blends of $CO_2$/Epoxy Cyclohexene Copolymer with Poly(vinyl phenol)

**Wei-Ting Du,[a] Yen-Ling Kuan,[a] and Shiao-Wei Kuo[a,b*]**

[a]Department of Materials and Optoelectronic Science, Center of Functional Polymers and Supramolecular Materials, National Sun Yat-Sen University, Kaohsiung 80424, Taiwan

[b]Department of Medicinal and Applied Chemistry, Kaohsiung Medical University, Kaohsiung, 807, Taiwan.

*Corresponding authors: E-mails: kuosw@faculty.nsysu.edu.tw

**Ligand LH**

A solution of 2,2-dimethyl-1,3-propanediamine in moderate MeOH was added dropwise to 2 equiv. of *o*-vanillin solution. The mixture was stirred for 4 h at room temperature and then the solvent was evaporated under vacuum to obtain a yellow powder. This synthesis was performed based on the report from the Williams group.[1] $^{1}$H NMR (500 MHz, $CDCl_3$, δ, ppm): 1.07 (s, 6H, $CC\mathbf{H}_3$), 3.49 (s, 4H, $NC\mathbf{H}_2$), 3.91 (s, 6H, $OC\mathbf{H}_3$), 6.77–6.95 (m, 6H, Ar**H**), 8.32 (s, 2H, N=C**H**), 14.15 (br, 2H, CO**H**). $^{13}$C NMR (125 MHz, $CDCl_3$, δ, ppm): 24.3 ($C\mathbf{C}H_3$), 36.3 ($\mathbf{C}$-$CH_3$), 56.2 ($O\mathbf{C}H_3$), 67.5 ($N\mathbf{C}H_2$), 114.3–123.5 (Ar**C**), 149.1 ($\mathbf{C}OCH_3$), 152.8 (**C**OH), 166.6 (N=**C**). FTIR (KBr, $cm^{-1}$): 1632 (aromatic C=C), 3450 (O–H).

**Catalyst $LZn_2(OAc)_2$**

A solution of $Zn(OAc)_2$·$2(H_2O)$ (2 equiv.) in MeOH was added to a solution of LH and then the mixture was stirred at room temperature overnight. Evaporation of the solvent vacuo provided a yellow powder. Washing with hexane, followed by crystallization from THF/hexane, yielded $LZn_2(OAc)_2$. This synthesis was performed based on a report from the Williams group.[1] $^{1}$H NMR (500 MHz, $CDCl_3$, δ, ppm): 1.03 (s, 6H, $CC\mathbf{H}_3$), 1.97 [s, $C(O)C\mathbf{H}_3$, 6H], 3.74 (s, 4H, $NC\mathbf{H}_2$), 3.86 (s, 6H, $OC\mathbf{H}_3$), 6.50–6.90 (m, 6H, Ar**H**), 8.04 (s, 2H, N=C**H**). $^{13}$C NMR (125 MHz, $CDCl_3$, δ, ppm): 23.3 [$C(O)\mathbf{C}H_3$], 25.4 (C-$\mathbf{C}H_3$), 35.5 ($\mathbf{C}CH_3$), 56.0 ($O\mathbf{C}H_3$), 75.0 ($N\mathbf{C}H_2$), 114.2–118.6 (Ar**C**), 127 ($\mathbf{C}OCH_3$), 150.9 (**C**-OH), 171.1 (N=**C**). FTIR (KBr, $cm^{-1}$): 1624 (aromatic C=C).

**Characterization**

$^{1}$H and $^{13}$C NMR spectra were recorded using an INOVA 500 MHz NMR spectrometer, with $CDCl_3$ as an external standard. Weight average ($M_w$) and number average ($M_n$) molecular weights and polydispersity indexes ($M_w/M_n$) were determined using a Water 510 gel permeation chromatograph. IR spectra were recorded using a Bruker Tensor 27 FTIR spectrophotometer and the conventional

crystal KBr disk method; 32 scans were collected at a spectral resolution of 4 $cm^{-1}$; a temperature-controlled compartment holder was used to record FTIR spectra at various temperatures, to maintain dry sample films. Glass transition temperatures were measured using a TA Q-20 differential scanning calorimeter; approximately 5 mg of sample was placed on a DSC sample pan and heated from 40 to 180 °C at a heating rate of 10 °C $min^{-1}$ under a $N_2$ atmosphere (50 mL $min^{-1}$). High-resolution solid state $^{13}C$ NMR spectroscopy was performed at 25 °C using a Bruker AVANCE III spectrometer operated at a resonance frequency of 100.62 MHz. The experiments were performed using the cross-polarization (CP)/magic-angle spinning (MAS)/high-power dipolar decoupling (DD) technique. The proton spin-lattice relaxation time in the rotating frame ($T_{1\rho}H$) was determined through carbon observation using a 90° $\tau$-spin lock pulse sequence prior to CP. The acquisition was performed at delay times ($s$) ranging from 0.1 to 30 ms with a contact time of 1.0 ms.

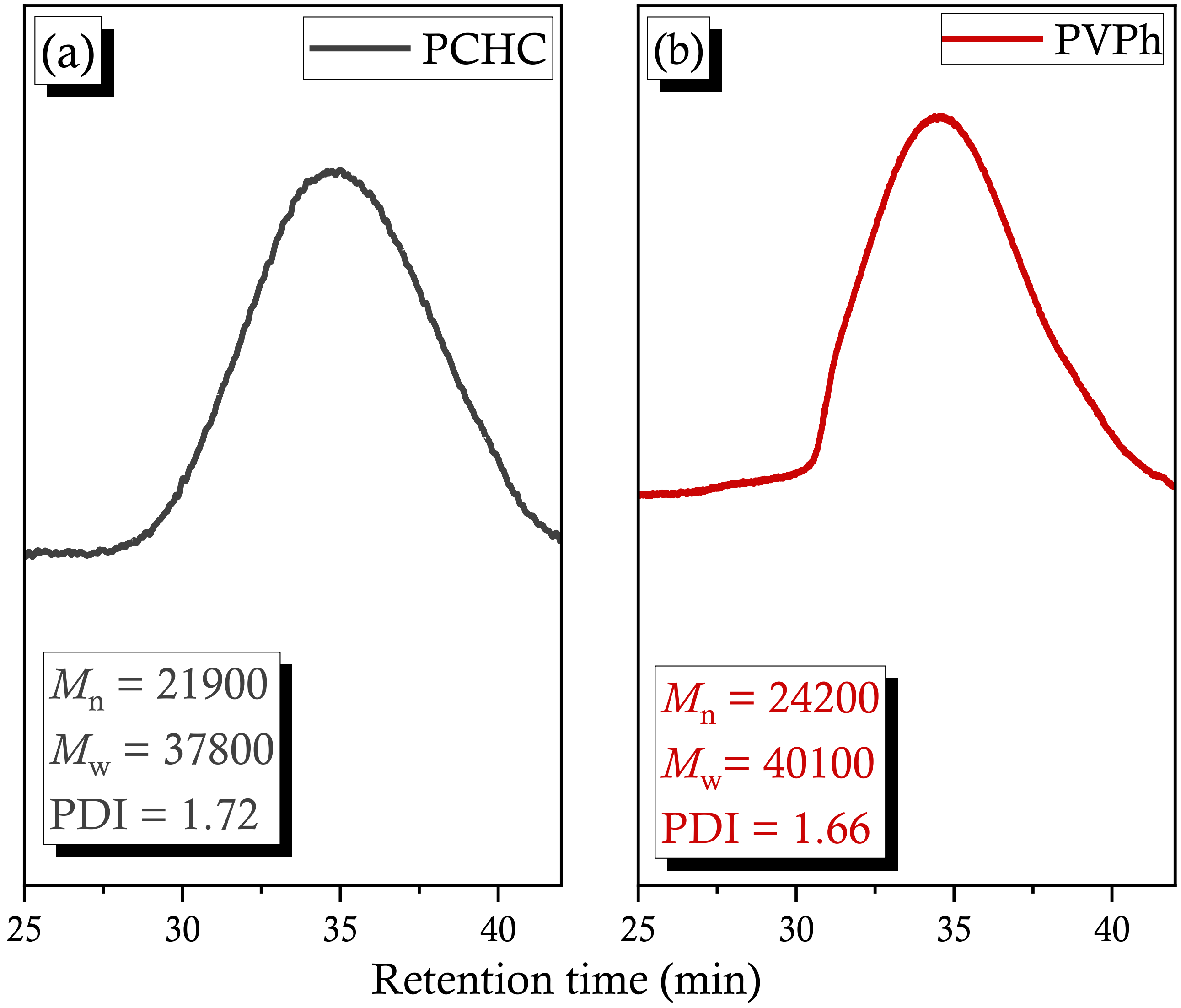


**Figure S1:** GPC analyses of (a) pure PCHC copolymer and (b) PVPh homopolymer

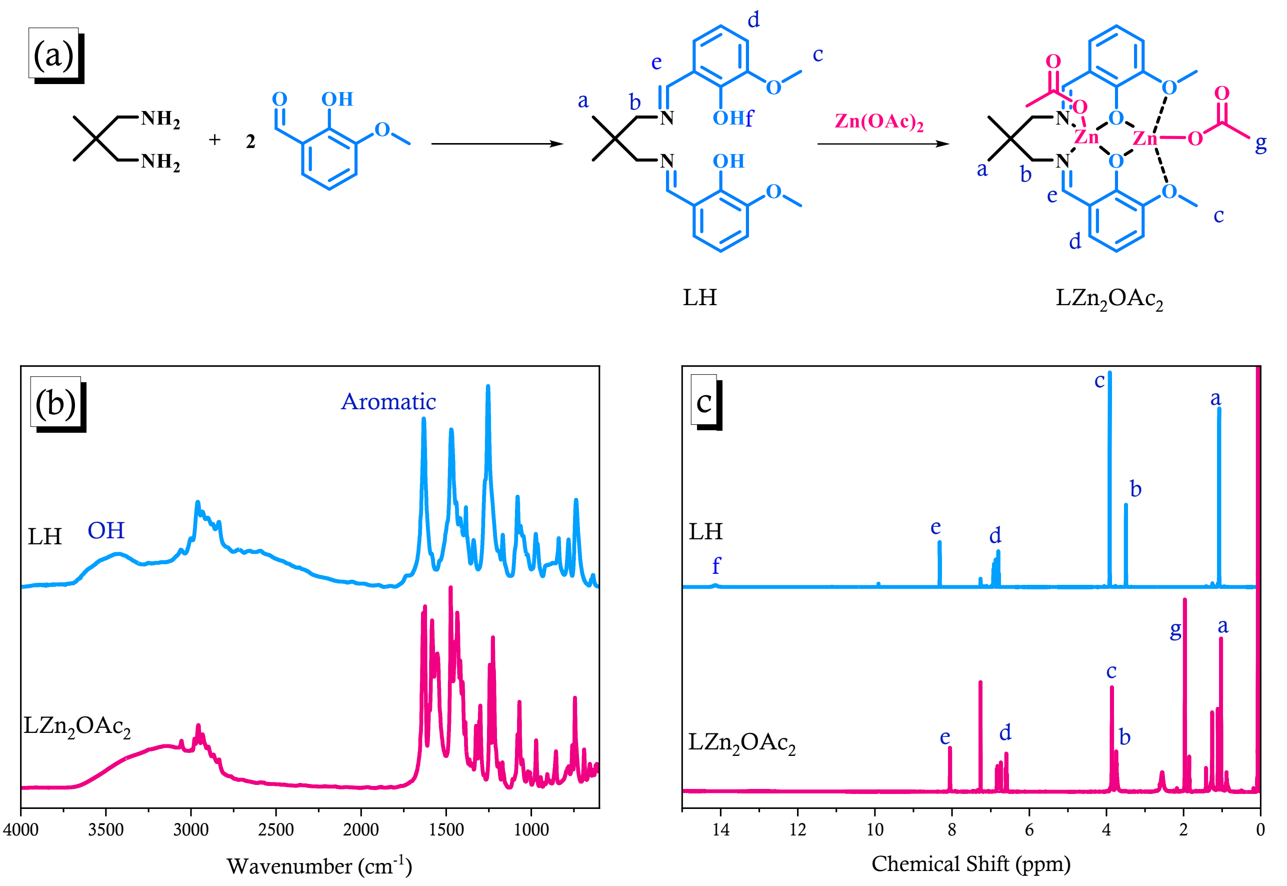


**Figure S2:** (a)The synthesis of LH and $LZn_2OAc_2$, (b) its related corresponding FTIR spectra and (c) $^1H$ NMR spectra

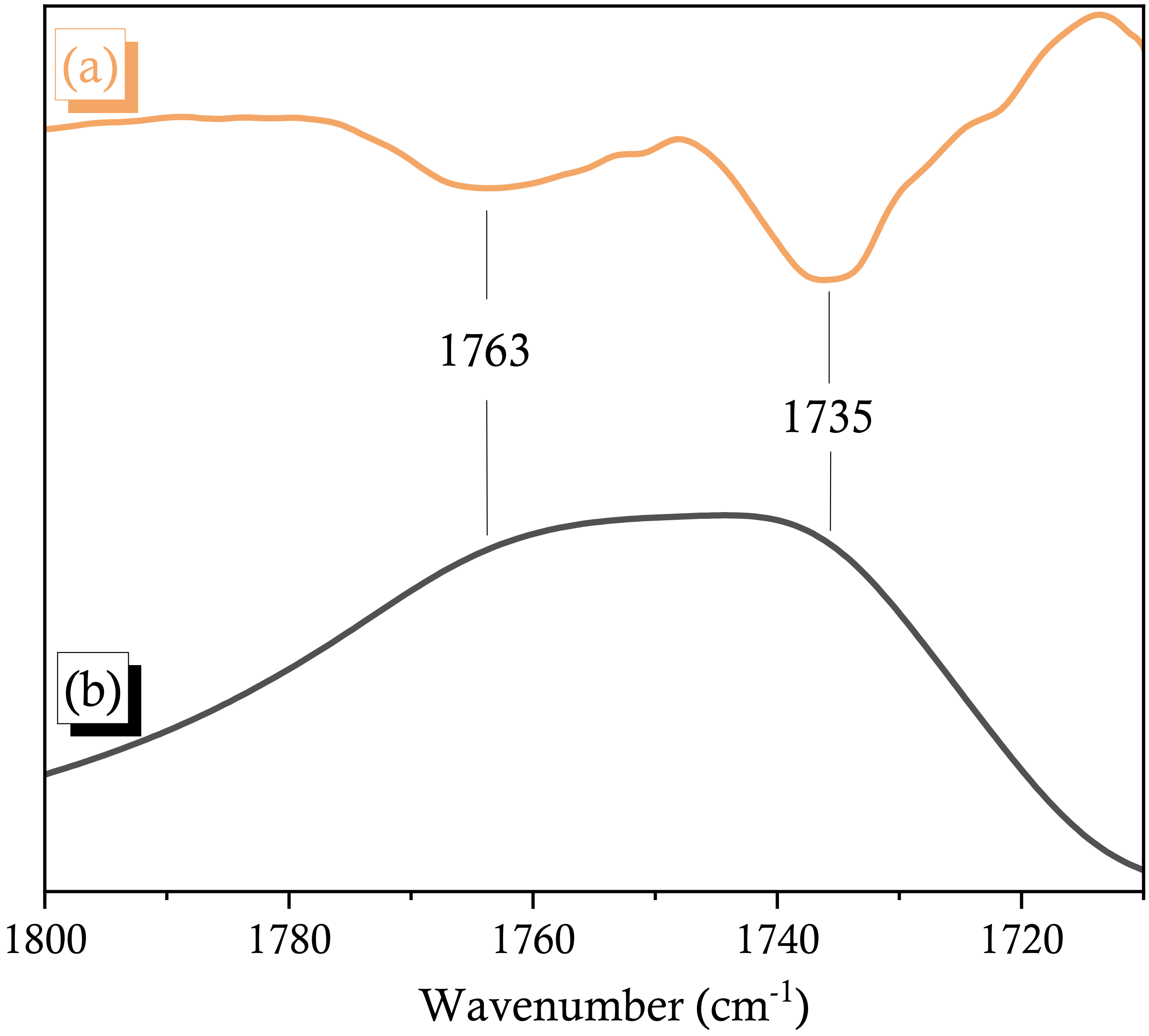


**Figure S3:** FTIR spectrum of C=O absorption and its second derivative spectrum of PCHC copolymer at 120 ˚C

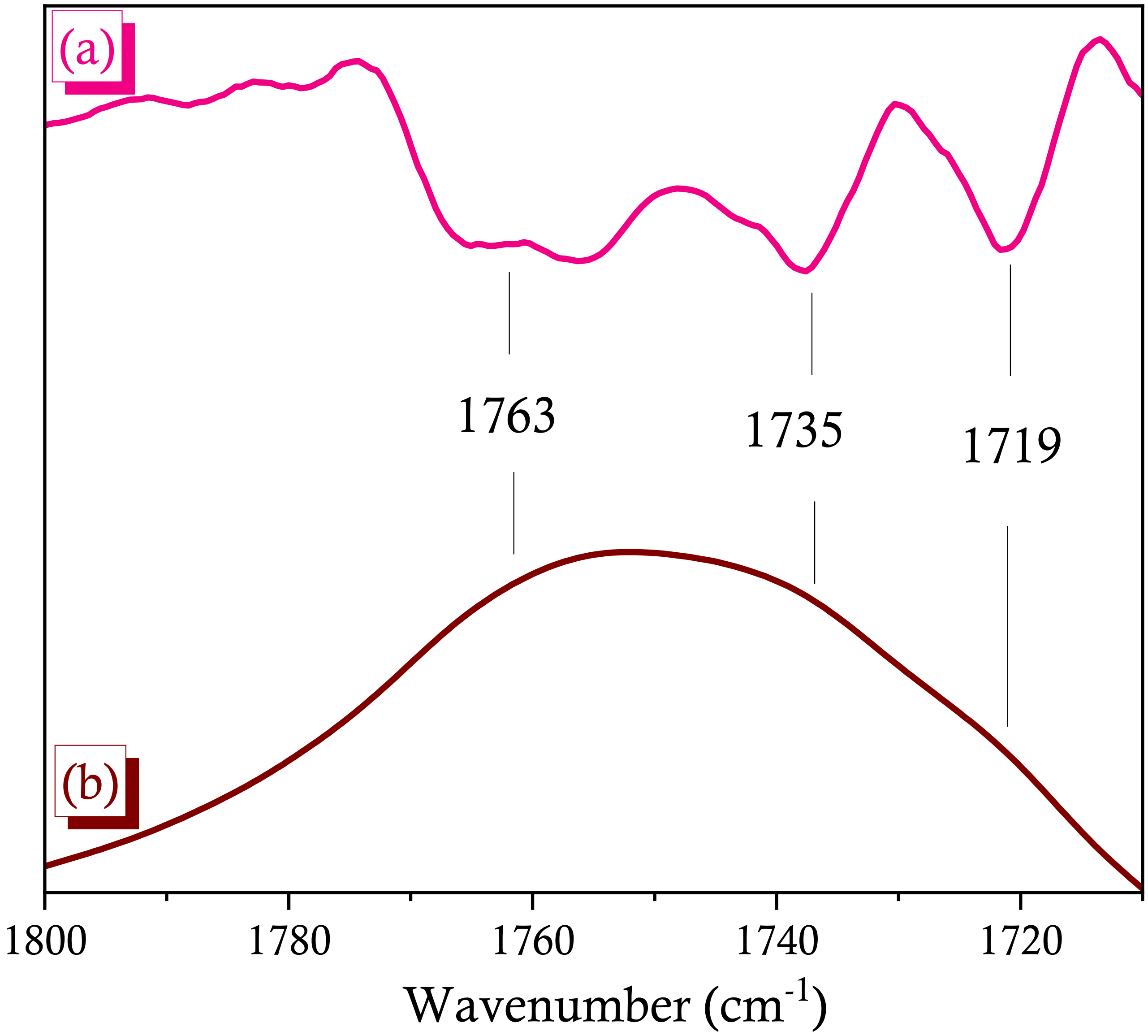


**Figure S4:** The IR spectrum of C=O absorption and its second derivative spectrum of PCHC-50 blend at 120 ˚C

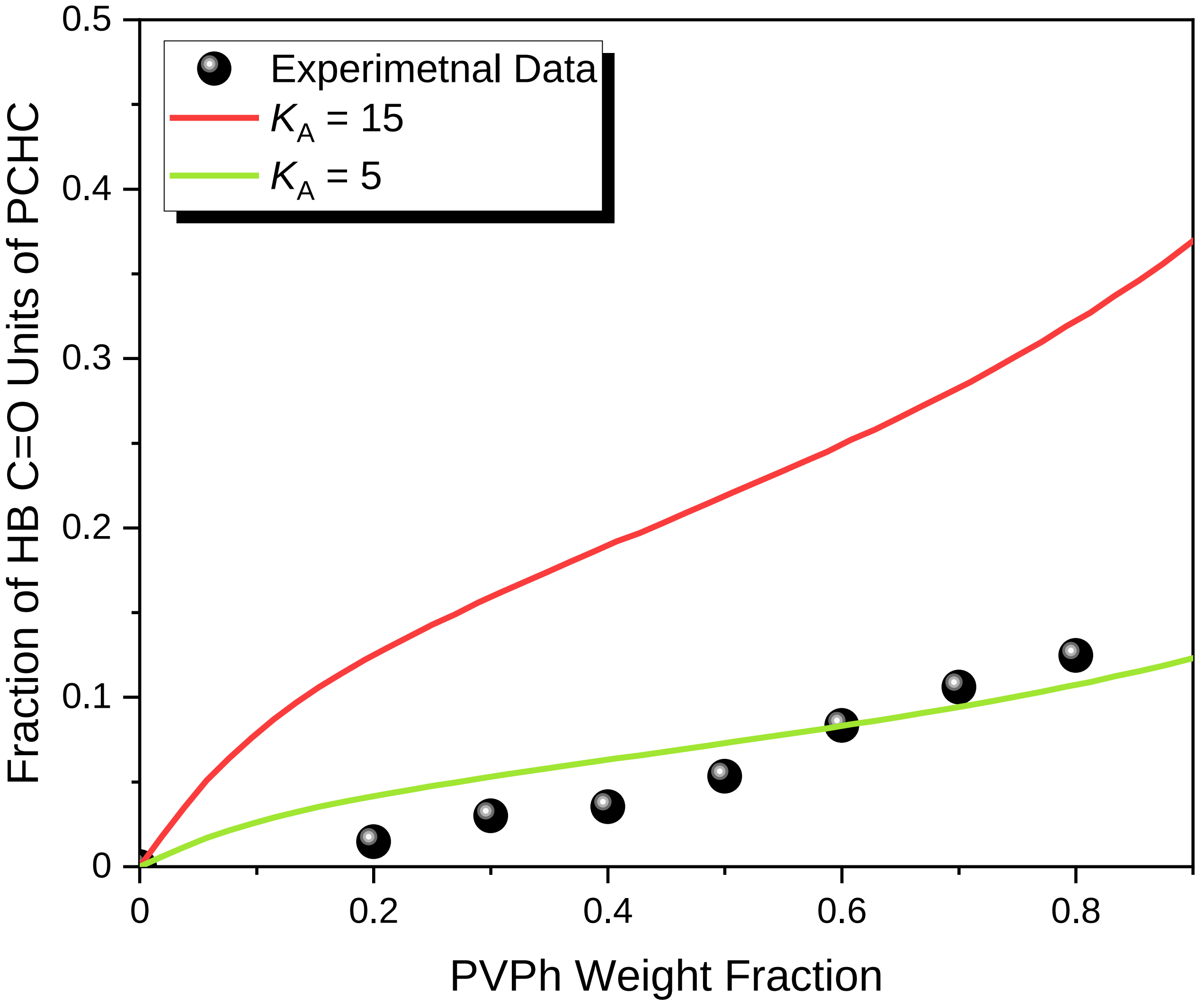


**Figure S5**: Experimental data about fraction of H-bonded C=O units of PCHC and predicted by PCAM

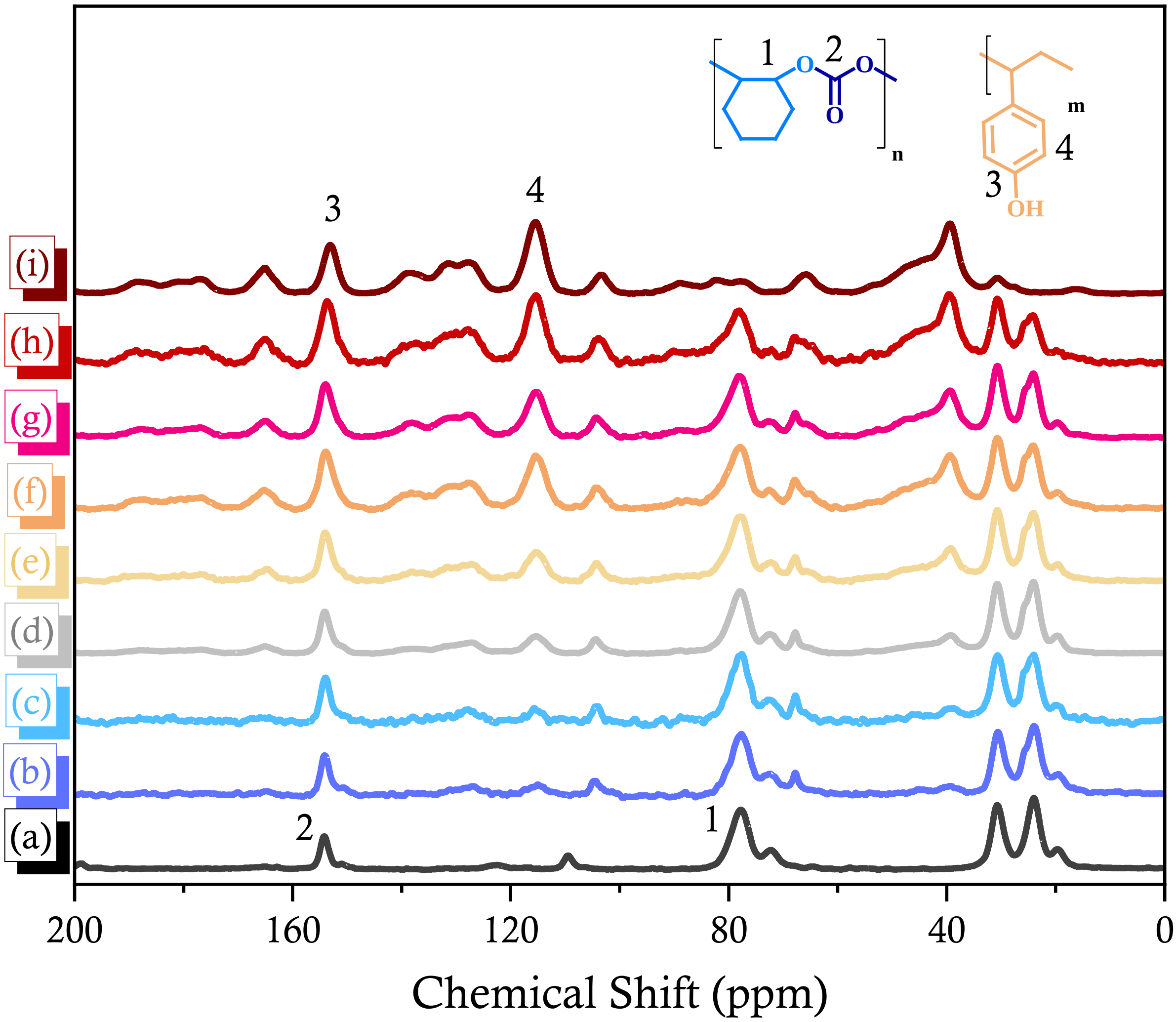


**Figure S6:** High-resolution solid-state $^{13}C$ NMR spectroscopy experiment was performed at 25 °C: (a) PCHC, (b) PCHC-80 (c) PCHC-70, (d) PCHC-60, (e) PCHC-50, (f) PCHC-40, (g) PCHC-30, (h) PCHC-20, (i) PVPh

## References

1. Thevenon, A.; Garden, J. A.; White, A. J. P.; Williams, C. K. Dinuclear Zinc Salen Catalysts for the Ring Opening Copolymerization of Epoxides and Carbon Dioxide or Anhydrides. *Inorg. Chem.* **2015**, *54*, 11906−11915.
